# Multi-omics analysis of a case of congenital microtia reveals aldob and oxidative stress associated with microtia etiology

**DOI:** 10.1186/s13023-024-03149-2

**Published:** 2024-05-27

**Authors:** Wenbo Liu, Yi Wu, Rulan Ma, Xinxi Zhu, Rui Wang, Lin He, Maoguo Shu

**Affiliations:** 1 The First Affiliated Hospital of Xi’an Jiao Tong University, No.277 Yanta West Road, Xi’an, Shaanxi 710061 China; 2 Department of Breast Surgery, Key Laboratory of Breast Cancer in Shanghai, Fudan University Shanghai Cancer Center, Shanghai, China; 3 Department of Surgical Oncology, The First Affiliated Hospital of Xi’an Jiao Tong University Medical College, Xi’an, Shaanxi China

**Keywords:** Microtia, Whole-exome sequencing, Label-free proteomics, Rare variants, Oxidative stress, ALDOB

## Abstract

**Background:**

Microtia is reported to be one of the most common congenital craniofacial malformations. Due to the complex etiology and the ethical barrier of embryonic study, the precise mechanisms of microtia remain unclear. Here we report a rare case of microtia with costal chondrodysplasia based on bioinformatics analysis and further verifications on other sporadic microtia patients.

**Results:**

One hundred fourteen deleterious insert and deletion (InDel) and 646 deleterious SNPs were screened out by WES, candidate genes were ranked in descending order according to their relative impact with microtia. Label-free proteomic analysis showed that proteins significantly different between the groups were related with oxidative stress and energy metabolism. By real-time PCR and immunohistochemistry, we further verified the candidate genes between other sporadic microtia and normal ear chondrocytes, which showed threonine aspartase, cadherin-13, aldolase B and adiponectin were significantly upregulated in mRNA levels but were significantly lower in protein levels. ROS detection and mitochondrial membrane potential (∆ Ψ m) detection proved that oxidative stress exists in microtia chondrocytes.

**Conclusions:**

Our results not only spot new candidate genes by WES and label-free proteomics, but also speculate for the first time that metabolism and oxidative stress may disturb cartilage development and this might become therapeutic targets and potential biomarkers with clinical usefulness in the future.

**Supplementary Information:**

The online version contains supplementary material available at 10.1186/s13023-024-03149-2.

## Backgrounds

Microtia-anotia (MIM: 600674) is a congenital malformation of the external and middle ear caused by the abnormal development of the first and second pharyngeal arch and the first sulcus at the embryonic stage. It may be accompanied by hearing loss, and also mandibular and facial soft tissue dysplasia [1]. Estimates of the incidence of microtia vary widely and range from 0.83 to 17.4 per 10,000 births [2]. Existing data indicate that inheritance is more likely in syndromic or familial microtia whereas multifactorial or polygenic causes are more probable in sporadic cases [3]. Since the fact that the ear is not only a functional organ, but also a crucial part of appearance, therefore, patients with microtia and their caretakers have suffered both physical and psychosocial burdens. Hence, to discovering the etiology of microtia has been a major topic in the plastic surgery field and public health.

About 40% of microtia patients present with other structural abnormalities as a syndrome [4], which involves chromosomal abnormalities and single-gene disorders. As goosecoid gene (*GSC*) has been identified as the most interesting candidate gene within the 14q32 chromosomal region, which was found to be connected with hemifacial microsomia and Goldenhar syndrome [5]. On the other hand, genetic variations that disrupt neural crest cells (NCCs) development can also be considered as an aetiology factor for sporadic cases of microtia. It is widely believed that *HOXA1* and *HOXA2* deficiency affecting the branchial arch development can cause microtia and other associated deformities. Meanwhile, some important pathways are also likely to affect the occurrence of microtia, such as bone morphogenetic proteins (BMPs), Wingless/INT (WNTs), fibroblast growth factors (FGFs) [6], and retinoic acid et al. [3]. Recently, epigenetic modifications such as DNA methylation and noncoding RNAs have been verified to affect the occurrence of microtia [7]. However, up till now, no mechanism mentioned oxidative stress and energy metabolism as the microenvironment’s imperative role in the pathogenesis of congenital microtia.

Here in this report, we study a rare case of congenital microtia with costal-chondrodysplasia based on the whole-exome sequencing, several candidate genes and related pathways are identified and new CNVs are additionally spotted. Secondly, label-free proteomics is used to assess microtia-associated protein changes. Altogether, we find that genes and the significant pathways are significantly related to the oxidative stress and energy metabolism process. Then real-time PCR and IHC show that ALDOB, ADIPOQ, CDH13 and TASP1 are probable pathogenic genes and ROS analysis and JC-1 assay both prove that oxidative stress exists in microtia. The expectation is that more insights into the molecular circuitry of microtia could be gained and more potential biomarkers and amelioration of microenvironment during embryonic development should benefit disease treatment and prevention.

## Results

### Clinical Report

**Fig. 1:**
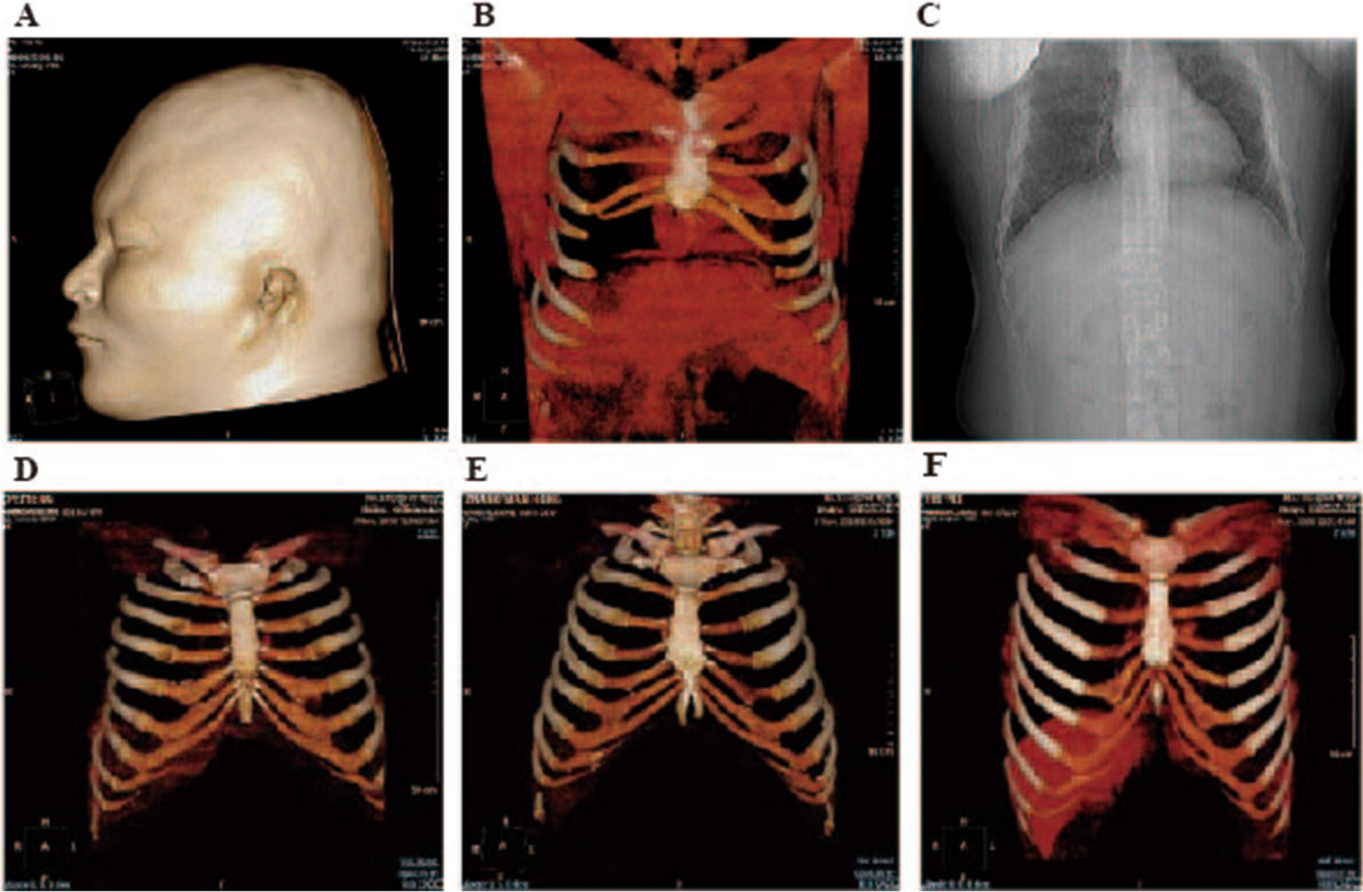
The phenotype of the patients and family members **A** Three-dimensional (3D) Computed Tomography (CT) reconstructions of the patient head showed left-side microtia **B** 3D-CT reconstruction of the patient costal area showed costal chondrodysplasia with sternal dysplasia. The costal cartilage is missing on the 6–10 and not attached to the rib. **C** X-ray thorax shows no deformity of the vertebral column (D-F) 3D-CT results of the patient's father (**D**), mother **E** and sister’s **F** normal costal area

Here we present a sporadical microtia case of non-consanguineous parents. The male patient A, who was initially assessed at 20 years of age presenting a weight of 93 kg, height of 166 cm, head circumference of 59 cm, and showed left ear with third degree microtia [8]. Mother (C), father (D), and the sister (B) show no abnormality (Fig. 1D-F). None of the family members show psychomotor retardation. A is diagnosed as left-side congenital microtia. During our three-dimensional (3D) chest computed tomography (CT) (Fig. 1A, B), the patient has abnormal rib cartilage which led to the failure of the reconstruction of the outer ear because the lack of a well-sculptured cartilage framework. Meanwhile, the patient does not show abnormality in the vertebral column (Fig. 1 C). The patient showed no gestational exposure to specific medications according to the mother’s recall, so they did not perform prenatal diagnosis by karyotyping or chromosomal microarray analysis.

## WES

### ACMG Classification of single nucleotide polymorphisms (SNPs)

One hundred fourteen deleterious InDel and 646 deleterious SNPs are screened out (Table 1 & Supplementary Table 1 and 2). SNPs are also classified by American Society for Medical Genetics and Genomics (ACMG) system [9] (Supplementary Table 3), among which 2 are pathogenic: Potassium voltage-gated channel, KQT-like subfamily, member 4 (*KCNQ4*) and Lipopolysaccharide-responsive, beige-like anchor protein (*LRBA*). 8 are likely pathogenic: Leptin receptor (*LEPR*), G protein-coupled receptor 161 (*GPR161*), Superoxide dismutase-3 (*SOD3*), WD repeat-containing protein 36 (*WDR36*), Plasminogen (*PLG*), Protein kinase, DNA-activated, catalytic polypeptide (*PRKDC*), unc-45 myosin chaperone B (*UNC45B*), Threonine aspartase (*TASP1*).

**Table 1:** Variants in 4 samples identified by WES

	SNPs	INDELs
Total	139,736	16,763
Frequency	5148	2474
Function	1522	191
Exonic Function	1007	143
Deleterious	646	114

**Fig. 2:**
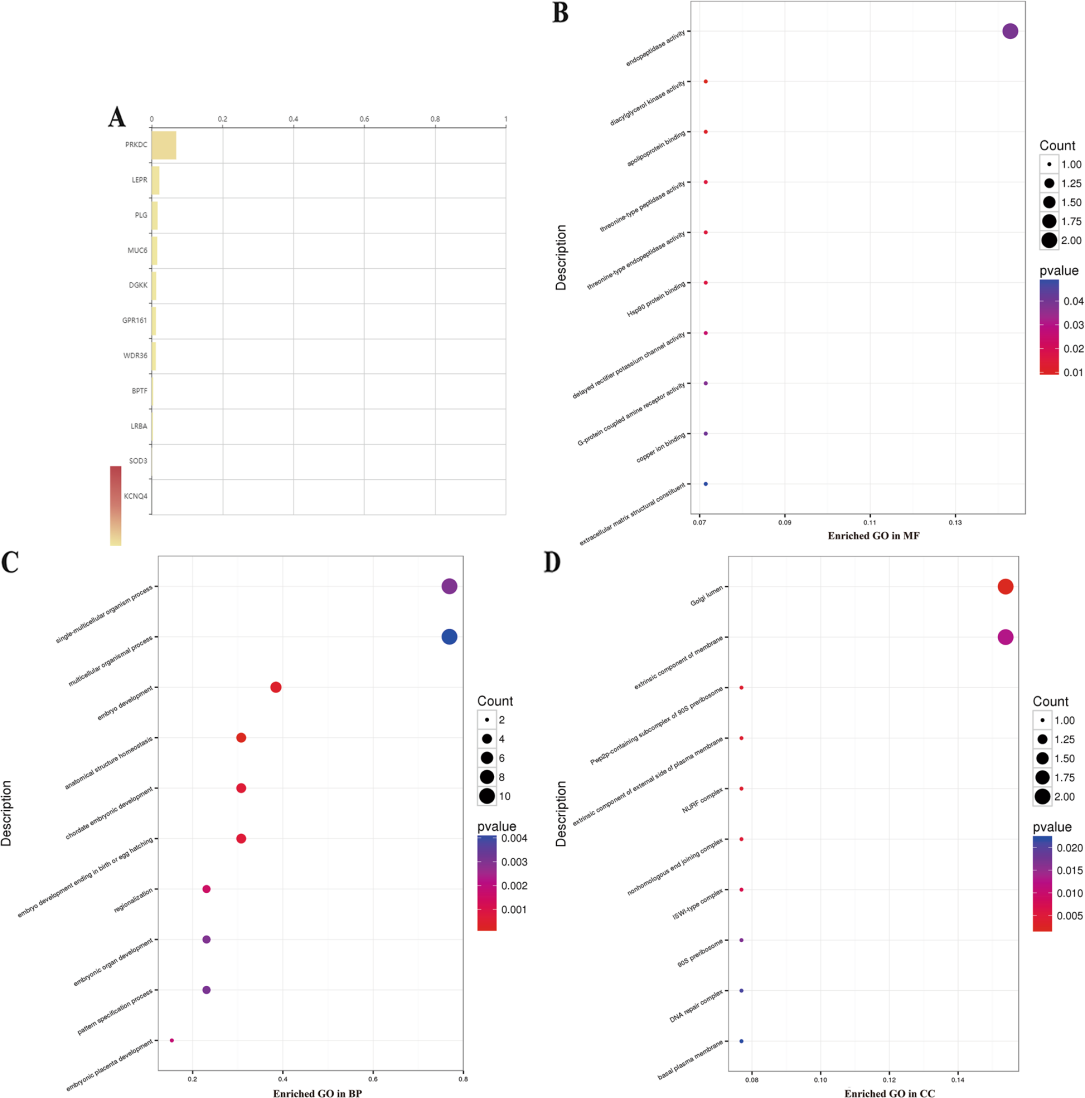
Candidate genes classified by the American Society for Medical Genetics and Genomics (ACMG) system and GO enrichments **A** Candidate genes are ranked according to how strongly they were associated with the disease by the ACMG system; The maximum correlation score is 1. **B** GO enrichment distribution map by molecular function (MF); **C** biological process (BP); **D** cell component (CC). The abscissa represents the proportion of enriched genes in the pathway to the total enriched genes, and the ordinate represents the name of the enriched GO term. The size of the dot represents the number of genes enriched in the term of the gene, and the color represents the *p*-value

These variants include 1 frameshift variant: *LEPR* (NM_001198687: exon17: c.2534delT:p.I845fs) and 6 missense variants: *KCNQ4* (NM_004700: exon4:c.C546G:p.F182L),*GPR161*(NM_001267613:exon2:.C802G:p.R268G),*SOD3*(NM_003102:exon2:c.C691G:p.R231G),*WDR36(*NM_139281:exon9:c.G1100A:p.G367D), *PLG* (NM_000301:exon15:c.G1858A:p.A620T), *UNC45B* (NM_001308281: exon15:c.G2071A:p.E691K). 1 stop gain: *LRBA* (NM_001199282: exon35:c.G5586A:p.W1862X) and 2 splice variants: *PRKDC* and *TASP1* (NM_001323603: exon7:c.98–1 > TA) (supplementary Table 3). Then, the following candidate genes ranked in descending order according to their relative impact on the development or severity of microtia: *PRKDC* > *LEPR* > *PLG* > *MUC6* > *DGKK* > *GPR161* > *WDR36* > *BPTF* > *LRBA* > *SOD3* > *KCNQ4* (Fig. 2A).

### Novel Variant or CNV Identification

The patient lacks a family history of genetic disease, that is, the pathogenic variants in the patient may be de novo mutations. Combining exome analysis, we identified de novo CNVs in espin pseudogene (*ESPNP)*, which is located in chromosomal 1p36.13, starting from 17,017,581 and ending at 17,030,650, the size is 13,070 bp, the CNV type is duplication and function as ncRNA exonic. Meanwhile, CNVs defined as possibly deleterious locate on chromosomal X, start from 1,571,538 to 2,139,310, with the size of 567,772, are acetyl serotonin methyltransferase (*ASMT*), dehydrogenase/reductase X-linked (*DHRSX*), acetyl serotonin O-methyltransferase like (*ASMTL*), A-kinase anchoring protein 17A (*AKAP17A*), P2Y receptor family member 8 (*P2RY8*). The CNV type is duplication (Supplementary Table 4). None of them have been reported before in microtia. Worth notice is that, the patient shares possibly deleterious CNVs on *ASMT*, *AKAP17A* and *DHRSX* with his father but not his mother (Supplementary Table 4) while his parents show no abnormality and this may be due to the incomplete penetrance.

### GO Analysis of the Mutant Genes

The GO analysis showed that the metabolism-, embryonic development-, communication-, and membrane-related genes were significantly enriched in the microtia. 11 pathways were significantly involved in molecular functions (MF) (Table 2), they are mainly about lipid metabolism and endopeptidase activity, some may involve copper ion binding and G-protein coupled amine receptor activity, while extracellular matrix structural constituent may also play a role in the formation of cartilage.

**Table 2:** GO enrichment of WES DEGs

GO enrichment	Description	GeneRatio	*p* value
**MF**	diacylglycerol kinase activity	1|14	0.00821591665313615
	apolipoprotein binding	1|14	0.0111879498220251
	threonine-type endopeptidase activity	1|14	0.0156304966499262
	threonine-type peptidase activity	1|14	0.0156304966499262
	Hsp90 protein binding	1|14	0.0163691167081804
	delayed rectifier potassium channel activity	1|14	0.0244600423482239
	G-protein coupled amine receptor activity	1|14	0.0375665588547606
	endopeptidase activity	2|14	0.0390655395157943
	copper ion binding	1|14	0.0404568248350441
	extracellular matrix structural constituent	1|14	0.049079337036385
**BP**	anatomical structure homeostasis	4|13	5.28452742366808e-05
	embryo development	5|13	0.000370965091163072
	chordate embryonic development	4|13	0.000636374602849953
	embryo development ending in birth or egg hatching	4|13	0.000660291996997452
	regionalization	3|13	0.00150553990717794
	embryonic placenta development	2|13	0.00180674229666208
	embryonic organ development	3|13	0.00299504282674374
	single-multicellular organism process	10|13	0.00299749453704538
	pattern specification process	3|13	0.00309374015426283
	multicellular organismal process	10|13	0.00409340218939154
	negative regulation of smoothened signaling pathway involved in dorsal/ventral neural tube patterning	1|13	0.00416911061950975
	placenta development	2|13	0.00453280606935473
	regulation of smoothened signaling pathway involved in dorsal/ventral neural tube patterning	1|13	0.00486240047983988
	developmental growth	3|13	0.00553454711966741
	negative regulation of immunoglobulin production	1|13	0.00555524478113389
	ectopic germ cell programmed cell death	1|13	0.00555524478113389
	negative regulation of fibrinolysis	1|13	0.00624764378589271
	multicellular organismal development	8|13	0.00670534554406182
	pro-B cell differentiation	1|13	0.00763110695510205
	negative regulation of gluconeogenesis	1|13	0.00763110695510205
	regulation of cell–cell adhesion mediated by cadherin	1|13	0.00763110695510205
	negative regulation of cellular senescence	1|13	0.00763110695510205
	developmental process involved in reproduction	3|13	0.00800778846276145
	anterior/posterior pattern specification	2|13	0.00863387296080742
	tissue homeostasis	2|13	0.00871510245775662
	diacylglycerol metabolic process	1|13	0.00901279208466399
	regulation of fibrinolysis	1|13	0.00901279208466399
	smoothened signaling pathway involved in dorsal/ventral neural tube patterning	1|13	0.00901279208466399
	V(D)J recombination	1|13	0.00970296853933572
	cell–cell adhesion mediated by cadherin	1|13	0.00970296853933572
	trophoblast giant cell differentiation	1|13	0.00970296853933572
	maintenance of gastrointestinal epithelium	1|13	0.0117708366023898
	mononuclear cell migration	1|13	0.0117708366023898
	negative regulation of cell aging	1|13	0.0117708366023898
	regulation of adenylate cyclase activity involved in G-protein coupled receptor signaling pathway	1|13	0.0124592397276797
	positive regulation of adenylate cyclase activity involved in G-protein coupled receptor signaling pathway	1|13	0.0124592397276797
	platelet activation	2|13	0.0125309851595471
	lymphoid progenitor cell differentiation	1|13	0.0131472001737192
	T cell lineage commitment	1|13	0.0131472001737192
	muscle cell cellular homeostasis	1|13	0.0131472001737192
	labyrinthine layer blood vessel development	1|13	0.0138347182014655
	system development	7|13	0.0143395474394994
	regulation of developmental growth	2|13	0.0148238150635097
	epithelial structure maintenance	1|13	0.0158946203824241
	removal of superoxide radicals	1|13	0.0158946203824241
	positive regulation of blood coagulation	1|13	0.0165803713437789
	response to copper ion	1|13	0.0165803713437789
	positive regulation of hemostasis	1|13	0.0165803713437789
	homeostatic process	4|13	0.016967030408323
	negative regulation of embryonic development	1|13	0.017265681189532
	dorsal/ventral neural tube patterning	1|13	0.0179505501798043
	positive regulation of coagulation	1|13	0.0179505501798043
	cellular response to oxygen radical	1|13	0.0179505501798043
	cellular response to superoxide	1|13	0.0179505501798043
	cellular detoxification	1|13	0.0179505501798043
	regulation of cellular senescence	1|13	0.0179505501798043
	single-organism developmental process	8|13	0.0180129743531054
	negative regulation of production of molecular mediator of immune response	1|13	0.0186349785745749
	fibrinolysis	1|13	0.0186349785745749
	cell differentiation involved in embryonic placenta development	1|13	0.0186349785745749
	double-strand break repair via nonhomologous end joining	1|13	0.0193189666336856
	negative regulation of smoothened signaling pathway	1|13	0.0193189666336856
	developmental process	8|13	0.0197627386746393
	response to superoxide	1|13	0.0200025146168371
	detoxification	1|13	0.0200025146168371
	response to oxygen radical	1|13	0.020685622783592
	placenta blood vessel development	1|13	0.020685622783592
	multicellular organismal homeostasis	2|13	0.0208612972403431
	protein kinase C-activating G-protein coupled receptor signaling pathway	1|13	0.0220505207054655
	cellular metabolic process	11|13	0.0221426060570995
	protein destabilization	1|13	0.0227323109790125
	regulation of cell aging	1|13	0.0227323109790125
	non-recombinational repair	1|13	0.02341366247302
	positive regulation of nucleobase-containing compound metabolic process	4|13	0.0240235159671877
	cell activation	3|13	0.0241209534081163
	growth	3|13	0.0241209534081163
	regulation of gluconeogenesis	1|13	0.0247750501577487
	in utero embryonic development	2|13	0.0251107391050237
	response to oxidative stress	2|13	0.025632500558246
	sensory perception	3|13	0.0267896454225642
	spleen development	1|13	0.026813847305463
	positive regulation of cellular biosynthetic process	4|13	0.0268856305460124
	developmental programmed cell death	1|13	0.0274925715535868
	positive regulation of nitrogen compound metabolic process	4|13	0.0278155315705463
	tissue development	4|13	0.0279724509399148
	neural tube patterning	1|13	0.0281708588313269
	positive regulation of biosynthetic process	4|13	0.0286056792465854
	somatic recombination of immunoglobulin gene segments	1|13	0.0295261235071052
	regulation of immunoglobulin production	1|13	0.0308796433943079
	thymus development	1|13	0.0308796433943079
	positive regulation of wound healing	1|13	0.0308796433943079
	negative regulation of cellular carbohydrate metabolic process	1|13	0.0315557496858115
	chaperone-mediated protein folding	1|13	0.0322314205522355
	somatic diversification of immunoglobulins	1|13	0.032906656250648
	negative regulation of blood coagulation	1|13	0.032906656250648
	labyrinthine layer development	1|13	0.032906656250648
	negative regulation of hemostasis	1|13	0.032906656250648
	reproductive structure development	2|13	0.0331073485123023
	negative regulation of cell-substrate adhesion	1|13	0.0335814570379775
	reproductive system development	2|13	0.0336920582065297
	negative regulation of coagulation	1|13	0.0342558231710161
	single organism reproductive process	3|13	0.0348975530532116
	response to gamma radiation	1|13	0.0356032525006954
	positive regulation of adenylate cyclase activity	1|13	0.0356032525006954
	cellular senescence	1|13	0.0356032525006954
	anatomical structure development	7|13	0.0356525240983885
	somatic diversification of immune receptors via germline recombination within a single locus	1|13	0.0362763162102293
	somatic cell DNA recombination	1|13	0.0362763162102293
	superoxide metabolic process	1|13	0.0369489462912592
	negative regulation of carbohydrate metabolic process	1|13	0.0369489462912592
	response to activity	1|13	0.0376211429998884
	positive regulation of fibroblast proliferation	1|13	0.0376211429998884
	tissue regeneration	1|13	0.0382929065920813
	system process	4|13	0.0393836856066387
	somatic diversification of immune receptors	1|13	0.040305601227635
	negative regulation of wound healing	1|13	0.0409756349109889
	O-glycan processing	1|13	0.0436514538084311
	positive regulation of cyclase activity	1|13	0.0443193308484787
	positive regulation of lyase activity	1|13	0.0443193308484787
	organ development	5|13	0.0444028345797032
	somitogenesis	1|13	0.044986777324194
	lens development in camera-type eye	1|13	0.044986777324194
	signal transduction involved in mitotic G1 DNA damage checkpoint	1|13	0.0456537934900342
	intracellular signal transduction involved in G1 DNA damage checkpoint	1|13	0.0456537934900342
	regulation of smoothened signaling pathway	1|13	0.0463203796003198
	reproductive process	3|13	0.0467819002284611
	signal transduction involved in mitotic cell cycle checkpoint	1|13	0.0469865359092334
	signal transduction involved in mitotic DNA damage checkpoint	1|13	0.0469865359092334
	signal transduction involved in mitotic DNA integrity checkpoint	1|13	0.0469865359092334
	sensory organ development	2|13	0.0472295316964494
	regulation of axon extension	1|13	0.0476522626708232
	signal transduction involved in DNA integrity checkpoint	1|13	0.0483175601389986
	signal transduction involved in DNA damage checkpoint	1|13	0.0483175601389986
	signal transduction involved in cell cycle checkpoint	1|13	0.0489824285675344
**CC**	Golgi lumen	2|13	0.00172997702043006
	NURF complex	1|13	0.00416911061950975
	extrinsic component of external side of plasma membrane	1|13	0.00416911061950975
	Pwp2p-containing subcomplex of 90S preribosome	1|13	0.00416911061950975
	nonhomologous end joining complex	1|13	0.00486240047983988
	ISWI-type complex	1|13	0.00693959775647524
	extrinsic component of membrane	2|13	0.0128202967744927
	90S preribosome	1|13	0.0165803713437789
	DNA repair complex	1|13	0.020685622783592
	basal plasma membrane	1|13	0.0220505207054655
	small-subunit processome	1|13	0.0220505207054655
	plasma membrane	7|13	0.0303012183980582
	platelet alpha granule lumen	1|13	0.032906656250648
	preribosome	1|13	0.0335814570379775
	basal part of cell	1|13	0.0335814570379775
	cell periphery	7|13	0.0337978466091075
	cytoplasmic membrane-bounded vesicle	3|13	0.0339842572828317
	nucleolar part	1|13	0.0416452367556718
	cytoplasmic vesicle	3|13	0.0428632874444538
	platelet alpha granule	1|13	0.0429831459494572
	secretory granule lumen	1|13	0.0429831459494572
	organelle lumen	6|13	0.0481583294146277

One hundred thirteen pathways of biological process (BP) were potentially related to the pathogenesis of congenital microtia since most are associated with embryo development related process (Table 2), top 10 among which are single-multicellular organism process (10/13), multicellular organismal process (10/13), multicellular organismal development (8/13), single-organism developmental process (8/13), developmental process (8/13), system development (7/13), embryo development (5/13), anatomical structure homeostasis (4/13), chordate embryonic development (4/13).

Twenty-two pathways of cell component (CC) reflect that the endocrine system may play crucial roles in auricle development (Table 2), among which are plasma membrane (7/13), cell periphery (7/13), organelle lumen (6/13), cytoplasmic membrane-bounded vesicle (3/13), cytoplasmic vesicle (3/13) etc. (Table 2).

## Label-free proteomics

### Identification of proteins involved in microtia

Forty-nine were significantly differentially expressed between the patient and sister (Table 3). Among those are 28 upregulated. Haptoglobin (HP) is increased by 10 times in group of A vs. B while in group of A vs. D the fold change is more than 6(Table 5). Protein S100-A9 (S100A9) in this comparison is 10 times higher, and repeat in group A vs. D by 4 times higher. Protein S100-A8 (S100A8) in the firs A vs. B and the A vs. D group (Table 5) is also upregulated with about 4 times and 2 times respectively. 21 are downregulated including Cadherin-13 (CDH13) and Adiponectin (ADIPOQ), Sex hormone-binding globulin (SHBG), Coagulation factor XIII A chain (F13A1), Cell surface glycoprotein MUC18 (MCAM), this trend remains in the group A vs. D and the latter two remain the same in group A vs. C (Table 4).

**Table 3:** Significantly differently expressed proteins (DEPs) between the patient(A) and the sister (B) / father (C) /mother (D)

Protein change	Protein IDs	Gene Name	A/B	*P* value
Up	sp|P19652|	ORM2	52.0098	0.01572
	sp|P0DJI8|	SAA1	13.5716	0.00025
	sp|P00738|	HP	12.3553	8.40E-05
	sp|P68871|	HBB	10.4074	0.00015
	sp|P06702|	S100A9	10.1012	0.01711
	sp|P02656|	APOC3	7.6401	0.00065
	sp|P02652|	APOA2	6.05155	0.00051
	sp|P05062|	**ALDOB**	5.41239	8.40E-06
	sp|P35542|	SAA4	5.00694	0.01798
	sp|O95497|	VNN1	4.68322	0.00692
	sp|P05109|	S100A8	4.03479	9.60E-05
	sp|Q92954|	PRG4	3.71812	7.30E-05
	sp|P02654|	APOC1	3.26363	0.00022
	sp|P01833|	PIGR	3.17335	0.0655
	sp|P49913|	CAMP	2.90301	0.00701
	sp|O00187|	MASP2	2.82309	9.80E-05
	sp|P0DOX3|	IGHD	2.57198	0.00114
	sp|O75636|	FCN3	2.55084	0.0001
	sp|P02743|	APCS	2.4263	0.00432
	sp|P32119|	PRDX2	2.39406	0.01119
	sp|P02649|	APOE	2.37657	0.00177
	sp|P0DOX2|	IGA2	2.26033	0.00604
	sp|P05090|	APOD	2.12998	0.00026
	sp|P18428|	LBP	2.08248	0.00012
	sp|P11226|	MBL2	2.06612	0.00122
	sp|O14791|	APOL1	2.05657	0.01408
	sp|P14780|	MMP9	2.04848	0.07726
	sp|P02655|	APOC2	2.0143	0.01408
Down	sp|P00747|	PLG	0.92449	0.22958
	sp|Q12805|	EFEMP1	0.49929	0.00435
	sp|P41222|	PTGDS	0.48757	0.0236
	sp|P08294|	SOD3	0.48261	0.02906
	sp|P10124|	SRGN	0.47759	0.15113
	sp|O43866|	CD5L	0.46049	0.00686
	sp|P01860|	IGHG3	0.45764	0.01141
	sp|P04196|	HRG	0.43655	0.00048
	sp|Q9NQ79|	CRTAC1	0.42915	0.00601
	sp|P01619|	IGKV3-20	0.42892	0.03818
	sp|A2NJV5|	IGKV2-29	0.4177	0.37041
	sp|P01871|	IGHM	0.39845	0.00207
	sp|P14151|	SELL	0.39284	0.00107
	sp|P23142|	FBLN1	0.38024	2.50E-05
	sp|P13591|	NCAM1	0.37942	0.01541
	sp|P01701|	IGLV1	0.36609	0.02552
	sp|P20742|	PZP	0.35211	0.02747
	sp|P55290|	**CDH13**	0.35024	0.02803
	sp|P43121|	MCAM	0.31941	0.04769
	sp|P00488|	F13A1	0.27724	0.00982
	sp|Q15848|	**ADIPOQ**	0.26241	0.04391
	sp|P04278|	SHBG	0.08718	3.40E-05

**Table 4:** Top 5 over-represented GO terms mapped to each category of enriched proteins (A vs B)

Protein change	Protein IDs	Gene Name	A/C	*P* value
Up	sp|P01009|	SERPINA1	13.4337	0.02449
	sp|P08294|	SOD3	10.0086	0.00044
	sp|P0DOX3|	IGHD	7.1257	0.00028
	sp|P01833|	PIGR	4.09544	0.01739
	sp|P68871|	HBB	4.07755	0.00034
	sp|P05062|	ALDOB	3.87038	1.33E-06
	sp|O95497|	VNN1	3.68009	0.00859
	sp|P16930|	FAH	3.5907	0.02486
	sp|P00915|	CA1	3.46278	1.25E-05
	sp|P02763|	ORM1	3.3588	0.00662
	sp|P0DJI8|	SAA1	2.97043	0.00117
	sp|P04040|	CAT	2.50931	0.03291
	sp|P02787|	TF	2.35841	5.48E-05
	sp|P05019|	IGF1	2.13296	0.01742
	sp|Q15485|	FCN2	2.12113	0.06902
	sp|P02652|	APOA2	2.11531	0.00303
	sp|P32119|	PRDX2	2.11019	0.01204
	sp|P19652|	ORM2	2.01404	0.03307
Down	sp|P00747	PLG	1.14564	0.03375
	sp|P43121	MCAM	0.49291	0.07688
	sp|Q12805	EFEMP1	0.47778	0.00324
	sp|P00488	F13A1	0.39772	0.08146
	sp|P02647	APOA1	0.38069	1.33E-05
	sp|P20742	PZP	0.00088	0.00418

Among the 23 proteins that were significantly different between patient and mother (Table 4), 18 are upregulated. Immunoglobulin delta heavy chain (IGHD) repeat in three groups, but the highest fold change is in this group by more than 7 times. Apolipoprotein A-II (APOA2) increases in A vs. B by 6 times and in A vs. C by 2 times. Pantetheinase (VNN1) in the A vs. B and A vs. C is both about 4 times higher. Peroxiredoxin-2 (PRDX2) is upregulated in the first two groups by 2 times. Five proteins are downregulated, including EGF-containing fibulin-like extracellular matrix protein 1 (EFEMP1) repeats in the first two groups.

There are 27 significantly different proteins between the patient and the father (D), 20 are upregulated including and Serum amyloid A-1 protein (SAA1) by 21-times higher, Alpha-1-antitrypsin (SERPINA1) repeat in A vs. C and A vs D, the fold changes are 13 and 11 respectively. The same trend goes with Serotransferrin (TF), in A vs. C the fold change is about 2 while in A vs. D fold change is more than 10 times. Immunoglobulin alpha-2 heavy chain (IGA2) in the first and this group is increased by the same 2-times (Table 5)0.7 proteins are downregulated in this group.

**Table 5:** Top 5 over-represented GO terms mapped to each category of enriched proteins (A vs C)

Protein change	Protein IDs	Gene Name	A/D	*P* value
Up	sp|P19652|A1AG2_HUMAN	ORM2	22.519	0.00336
	sp|P0DJI8|SAA1_HUMAN; sp|P0DJI9|SAA2_HUMAN	SAA1	21.9293	0.00219
	sp|P01009|A1AT_HUMAN; sp|P20848|A1ATR_HUMAN	SERPINA1	11.8368	0.02544
	sp|P02787|TRFE_HUMAN;	TF	10.4678	9.30E-06
	sp|P00738|HPT_HUMAN	HP	6.65963	0.00012
	sp|P07327|ADH1A_HUMAN;	ADH1A	4.68652	0.16031
	sp|P0DOX3|IGD_HUMAN;	IGHD	4.57695	0.00042
	sp|P01833|PIGR_HUMAN	PIGR	4.2722	0.04831
	sp|P06702|S10A9_HUMAN	S100A9	4.05448	0.0299
	sp|P68871|HBB_HUMAN	HBB	3.19377	0.00056
	sp|P05062|ALDOB_HUMAN	ALDOB	2.85092	9.60E-06
	sp|P01624|KV315_HUMAN;	IGKV3-15	2.58108	0.0027
	sp|P0DOX2|IGA2_HUMAN	IGA2	2.4137	0.00393
	sp|P01876|IGHA1_HUMAN	IGHA1	2.35757	0.00473
	sp|Q7Z7G0|TARSH_HUMAN	ABI3BP	2.33537	0.03622
	sp|P02042|HBD_HUMAN;	HBD	2.28108	0.04828
	sp|P16930|FAAA_HUMAN	FAH	2.17839	0.06318
	sp|P02741|CRP_HUMAN	CRP	2.09923	0.01983
	sp|P06331|HV434_HUMAN;	IGHV4-34	2.0825	0.11877
	sp|P05109|S10A8_HUMAN	S100A8	2.02529	0.00183
Down	sp|P00488|F13A_HUMAN	F13A1	0.48746	0.08864
	sp|Q14515|SPRL1_HUMAN	SPARCL1	0.47458	0.21468
	sp|P24592|IBP6_HUMAN	IGFBP6	0.4501	0.01221
	sp|P55290|CAD13_HUMAN	CDH13	0.43232	0.05011
	sp|P43121|MUC18_HUMAN	MCAM	0.31112	0.02734
	sp|Q15848|ADIPO_HUMAN	ADIPOQ	0.29045	0.17271
	sp|P04278|SHBG_HUMAN;	SHBG	0.14924	3.00E-05

Ten proteins that overlap between three groups are Alpha-1-acid glycoprotein 2 (ORM2), SAA1, IGHD, Polymeric immunoglobulin receptor (PIGR), Hemoglobin subunit beta (HBB), Fructose-bisphosphate aldolase B (ALDOB), SOD3, Plasminogen (PLG), F13A1, MCAM. Especially for ORM2 is even for more than 52 times up-expressed in A vs. B and 22 times higher in A vs. D group. SOD3 is halved in the comparison between the patient and the sister, while upregulated more than 10 times in the group between the patient and the mother but remains unchanged in comparison of patient to father. PLG appears the same in three groups, of which fold-change is about 1. F13A1 and MCAM are downregulated in three groups.

When compare the proteomics results to the WES SNPs genes, there are overlapped results: Mannan-binding lectin serine protease 2, *MASP2* (chromosome 1 11,105,527 C > T exonic, missense SNVs) and Ficolin (collagen/fibrinogen domain-containing lectin) 2, (*FCN2)* (NM_004108: exon5: c.429 + 1G > A; NM_015837: exon4: c.315 + 1G > A).

### GO enrichment analysis

The top 5 over-represented GO terms, including MF, BP, and CC categories of the common up and downregulated proteins were summarized in Tables 6, 7 and 8. GO level 2 analysis shows that proteins in three groups in the BP were principally enriched by biological process, biological regulation, single-organism process, and cellular process. In the MF, proteins were mainly enriched in molecular function, protein binding, ion binding, receptor binding and so on. In the CC, proteins were mainly enriched in cellular component, cell part, organelle, extracellular region part, and extracellular region.

**Table 6:** Top 5 over-represented GO terms mapped to each category of enriched proteins (A vs D)

Category	GO ID	Name	Count
BP	GO:0008150	Biological process	45
	GO:0065007	biological regulation	45
	GO:0044699	single-organism process	45
	GO:0050789	regulation of biological process	45
	GO:0009987	cellular process	45
MF	GO:0003674	Molecular function	45
	GO:0005488	binding	44
	GO:0005515	protein binding	41
	GO:0043167	ion binding	27
	GO:0043169	cation binding	20
CC	GO:0005575	Cellular component	45
	GO:0044421	extracellular region part	45
	GO:0043226	organelle	45
	GO:0044464	cell part	45
	GO:0005623	cell	45
	GO:0005576	extracellular region	45
	GO:0043227	membrane-bounded organelle	45

**Table 7:** Proteins GO terms Enrichment level 2

Category	GO ID	Name	Count
BP	GO:0008150	Biological process	20
	GO:0050896	response to stimulus	20
	GO:0009987	cellular process	20
	GO:0044699	single-organism process	20
	GO:0044763	single-organism cellular process	20
MF	GO:0003674	Molecular function	20
	GO:0005488	binding	19
	GO:0005515	protein binding	19
	GO:0043167	ion binding	13
	GO:0003824	catalytic activity	11
CC	GO:0005575	Cellular component	20
	GO:0044421	extracellular region part	20
	GO:0043226	organelle	20
	GO:0044464	cell part	20
	GO:0005623	cell	20
	GO:0005576	extracellular region	20
	GO:0043227	membrane-bounded organelle	20

**Table 8:** Top 5 over-represented GO terms mapped to each category of enriched proteins (A vs D)

Category	GO ID	Name	Count
BP	GO:0008150	Biological process	20
	GO:0044699	single-organism process	20
	GO:0050789	regulation of biological process	20
	GO:0065007	biological regulation	20
	GO:0009987	cellular process	20
MF	GO:0003674	Molecular function	20
	GO:0005488	binding	20
	GO:0005515	protein binding	19
	GO:0043167	ion binding	14
	GO:0005102	receptor binding	10
CC	GO:0005575	Cellular component	20
	GO:0044464	cell part	20
	GO:0043226	organelle	20
	GO:0044421	extracellular region part	20
	GO:0005623	cell	20
	GO:0005576	extracellular region	20

**Fig. 3:**
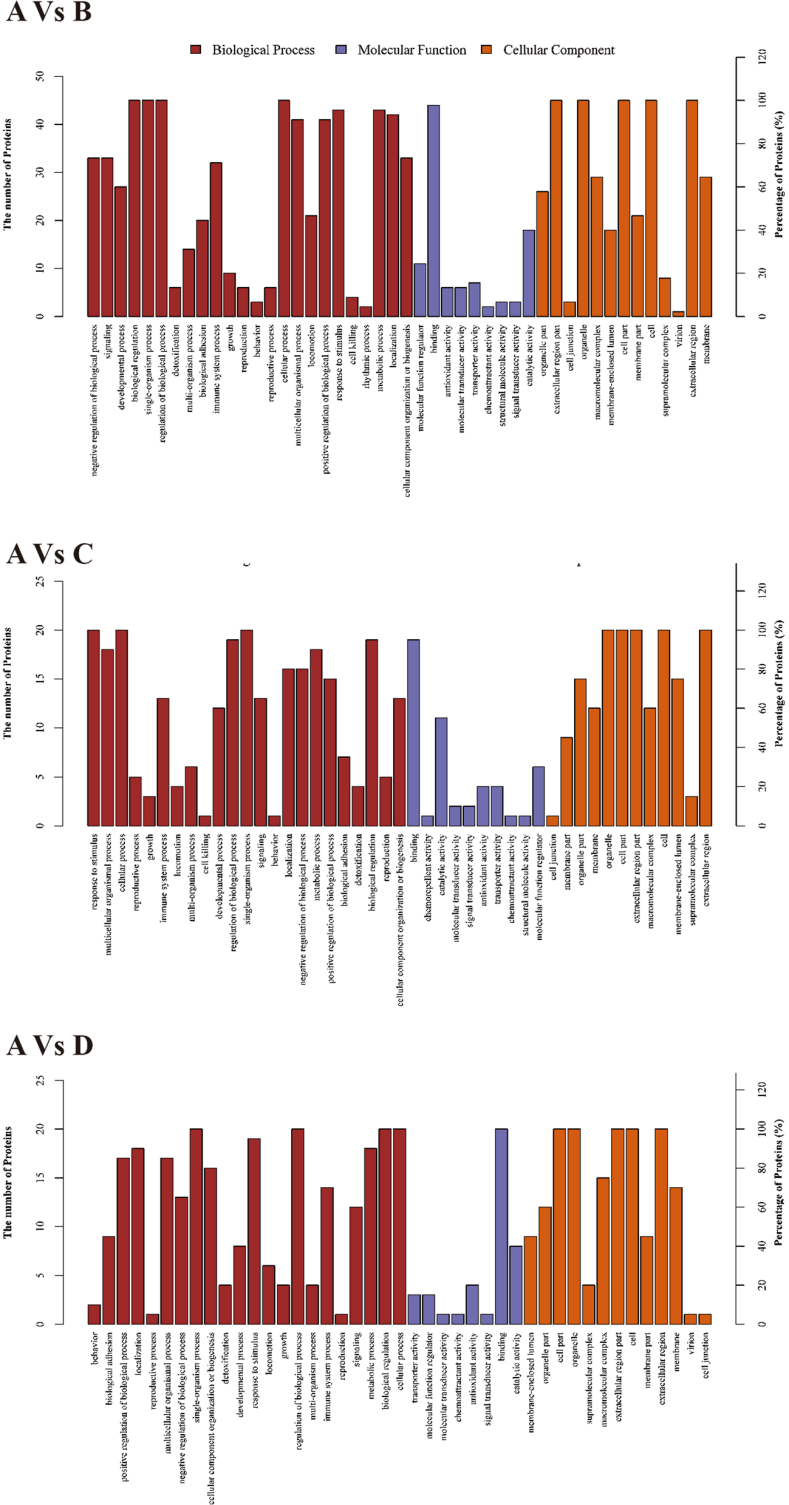
GO annotation of proteins level 2 Analysis **A**. Significantly different proteins when the patient **A** compared with sister **B** assigned by GO annotation; **B** patient **A** compared with mother **C**; **C** patient **A** compared with father **D**. Dark red means the biological process proteins contribute to, blue columns represent proteins’ molecular function, and orange means in which cellular component the proteins are active

When comparing the proteins of the patient to the database at level 2, GO enrichment showed that the proteins were mainly enriched in lipid metabolism-related GO terms and oxidative stress related areas (Table 9 and Fig. 3).

**Table 9:** Proteins GO terms Enrichment level 2

Group	GO-ID	Term	Category	FDR	*P*-Value
A Vs B	GO:0032372	negative regulation of sterol transport	P	0.0503	0.0000136
	GO:0032375	negative regulation of cholesterol transport	P	0.0503	0.0000136
	GO:0045833	negative regulation of lipid metabolic process	P	0.0787	0.000032
	GO:0034364	high-density particle lipoprotein	C	0.085	0.000046
	GO:0034382	chylomicron clearance remnant	P	0.0996	0.000134
A Vs C	GO:0018158	protein oxidation	P	0.761	0.000103
	GO:0006804	obsolete peroxidase reaction	P	0.952	0.000966
	GO:0016684	oxidoreductase activity, acting on peroxide as acceptor	F	0.952	0.000966
	GO:0004601	peroxidase activity	F	0.952	0.000966
	GO:0060205	cytoplasmic vesicle lumen	C	0.952	0.00113
A Vs D	GO:0005833	hemoglobin complex	C	0.535	0.000399
	GO:0031838	haptoglobin-hemoglobin complex	C	0.535	0.000399
	GO:0030492	hemoglobin binding	F	0.535	0.000399
	GO:0003014	renal system process	P	0.535	0.00073
	GO:0071682	endocytic vesicle lumen	C	0.535	0.00073
	GO:0005833	hemoglobin complex	C	0.535	0.000399

### KEGG pathway analysis

**Fig. 4:**
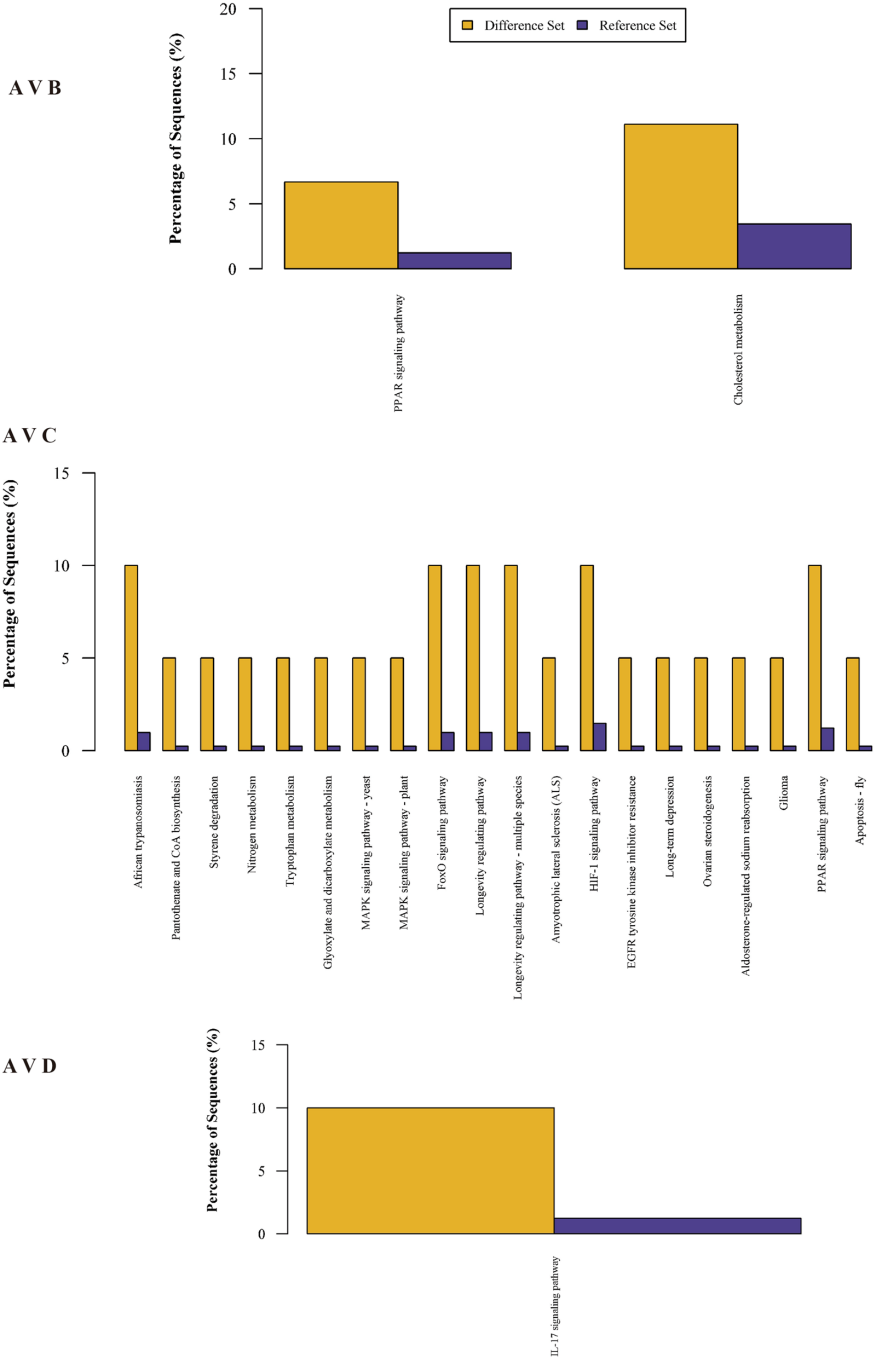
KEGG pathways enrichments of proteins. **A** The significantly different proteins in group patients **A** vs. sister **B** enrich in PPAR signaling and cholesterol metabolism pathway based on the KEGG database; **B**. The significantly different proteins in group A vs. father **C** enrich in many pathways, including FoxO, MAPK, PPAR, *etc.* **C**. The significantly different proteins in group A vs. mother **D** enrich in IL-17 signaling pathway

To gain an initial understanding of the role and function of the identified protein differences, we used KEGG pathway analysis to identify the biological pathways of the proteins that were significantly differentially expressed (> 1.5 fold upregulated or downregulated) between microtia and control samples. In Fig. 4, the KEGG classification results showed that genes were mainly enriched in PPAR signaling pathway, Cholesterol metabolism, Fox O signaling pathway, HIF-1 signaling pathway, IL-17 signaling pathway and so on (Fig. 4). In the group of A Vs. C (mother), the top 30 signaling pathway results showed that proteins are mainly enriched in Fox O signaling pathway, PPAR signaling pathway, HIF-1 signaling pathway, Amyotrophic lateral sclerosis (ALS), EGFR tyrosine kinase inhibitor resistance etc. (Fig. 4). When compared to his father, the IL-17 pathway is the only pathway that is significantly affected.

### PPI network analysis.

**Fig. 5:**
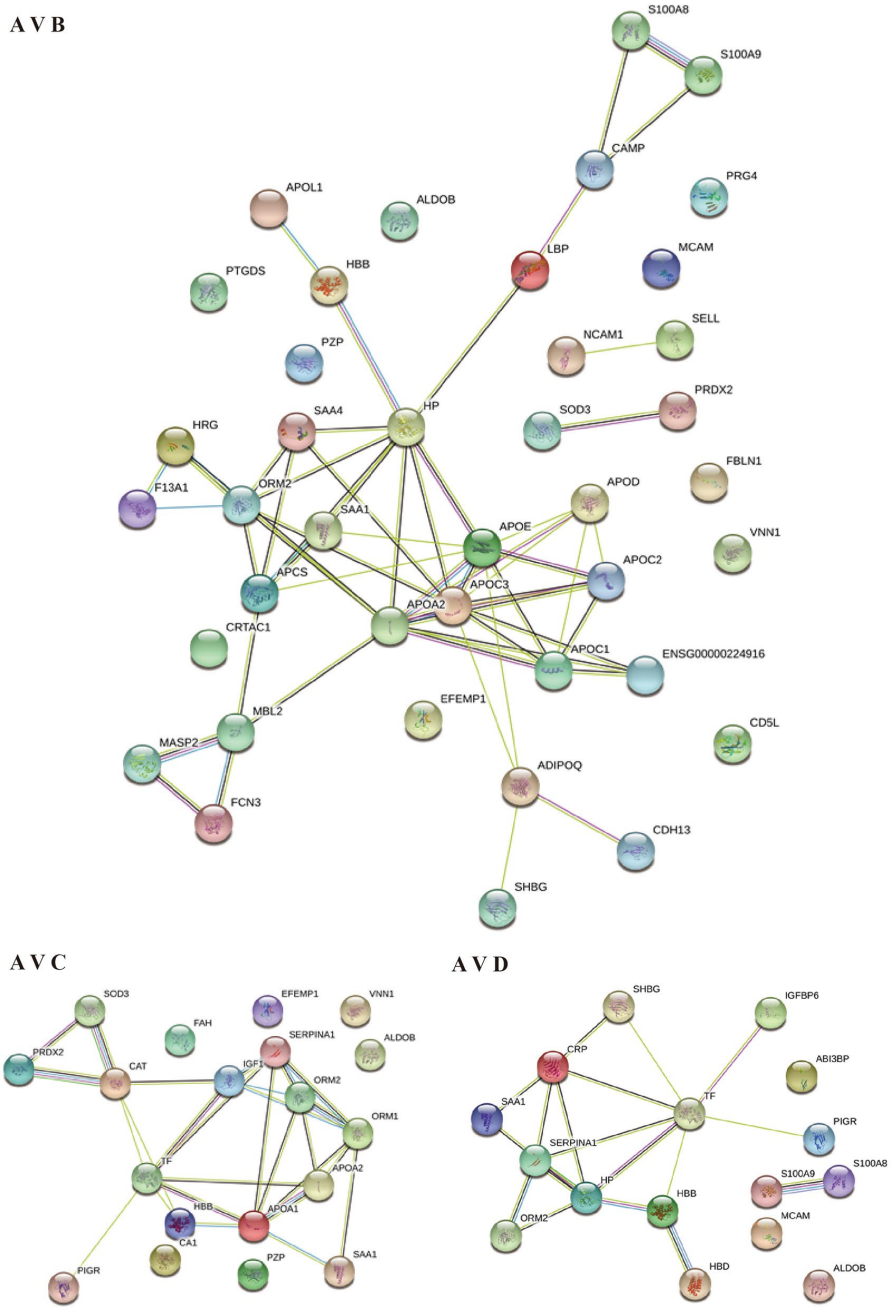
Protein–protein interaction (PPI) network. Network representation of signed PPIs shows significantly different proteins complexes and signaling pathways. Circles represent proteins while lines indicate molecular interactions

Proteins with similar expression patterns may have similar functions or participate in the same biological pathways, or be in adjacent regulatory positions in the pathways. To this end, to categorize the data based on functional similarity, we show the PPI network of differently expressed proteins. Some differently expressed proteins between the patient and the sister (Fig. 5 A V B) are correlated with each other, S100A8 is highly related to S100A9, APOA2, APOC1-3, APOE, and APOD are in the center of the STRING while SAA1, SAA4, HP, are closely related to the proteins mentioned above. SOD3 and PRDX2 repeat in the A vs. C.

### Further verifications on Other Microtia Patients

**Fig. 6:**
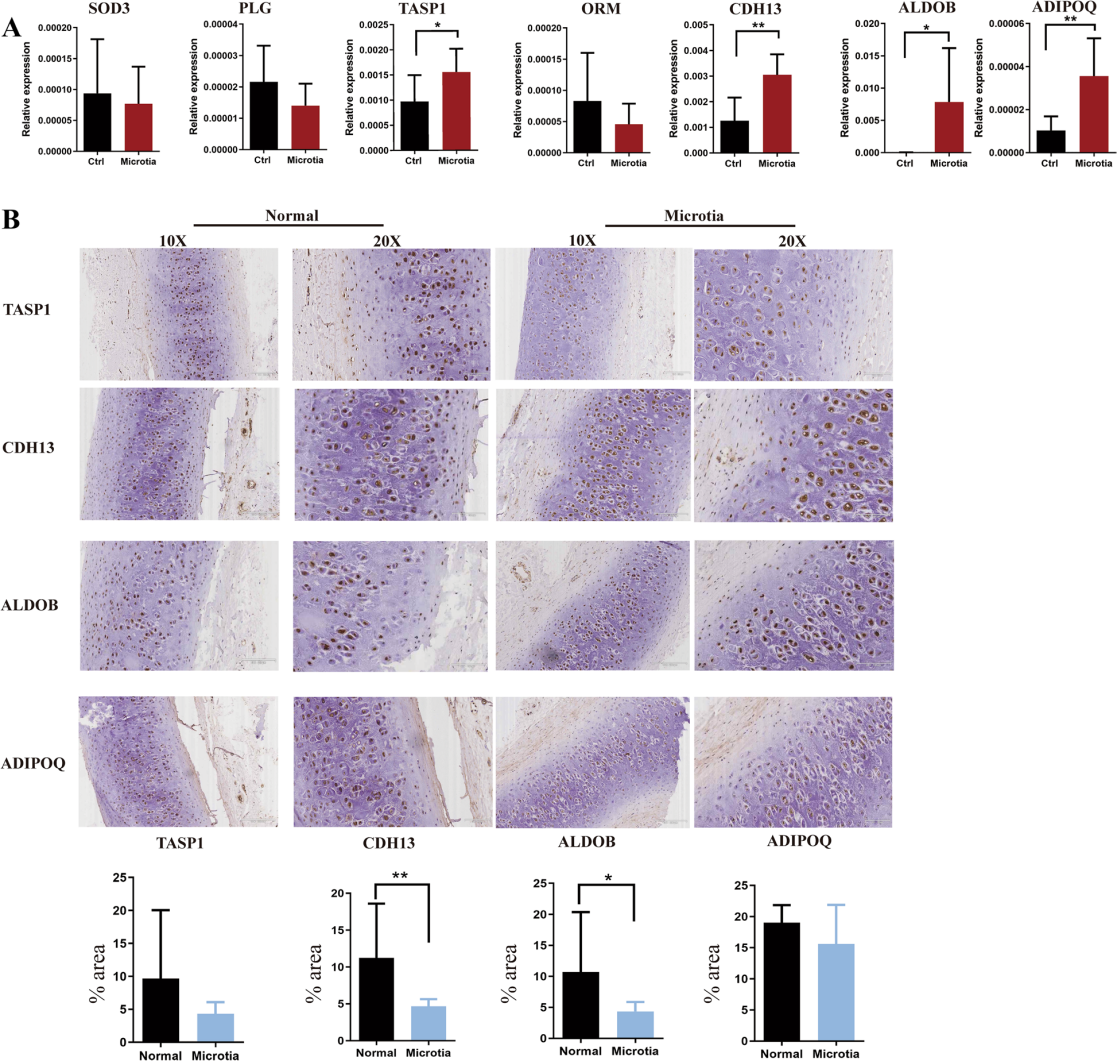
Candidate genes verifications between microtia patients and normal people. **A** Real-time PCR results mRNA level comparison. *TASP1*, *CDH13*, *ALDOB* and *ADIPOQ* are upregulated significantly in microtia patients’ ear chondrocytes when compared to the normal people as control. **B** IHC images and protein expression level comparison IHCs involved in the revealed markers of TASP1, CDH13, ALDOB and ADIPOQ are shown for microtia and normal ear cartilage. TASP1, CDH13 and ADIPOQ levels are significantly lower in the microtia group. Scale bars:10X: 200 μm, 20X: 100 μm.*p < 0.05, **p < 0.01. Error bars represent the mean ± SD of three independent experiments

As the variations of proteins between the patient and the controls don't necessary cause anomaly, further evaluation is necessary to investigate the potential roles of candidate genes identified in the multi-omic analysis. To accomplish this, we collected auricular cartilage tissues from individuals with microtia as well as from individuals who underwent rhinoplastic surgery or experienced accidental ear injuries. Firstly, we screened the differentially expressed genes. Real-time PCR results in Fig. 6 showed that *TASP1*, *CDH13*, *ALDOB* and *ADIPOQ* were significantly upregulated than the normal control group while the absolute expression level of ADIPOQ was not as high as other three, and the variance of ALDOB among different patients is higher. Secondly, we performed IHC of the four genes aforementioned on microtia patients and normal people as the control to verify the expression on protein level. The results showed that ALDOB, TASP1, and CDH13 were all significantly lower in the microtia group, while ADIPOQ showed no significance (Fig. 6).

### ROS Detection and mitochondrial membrane potential (∆Ψm) detection proved that oxidative stress exists in microtia chondrocytes

**Fig. 7:**
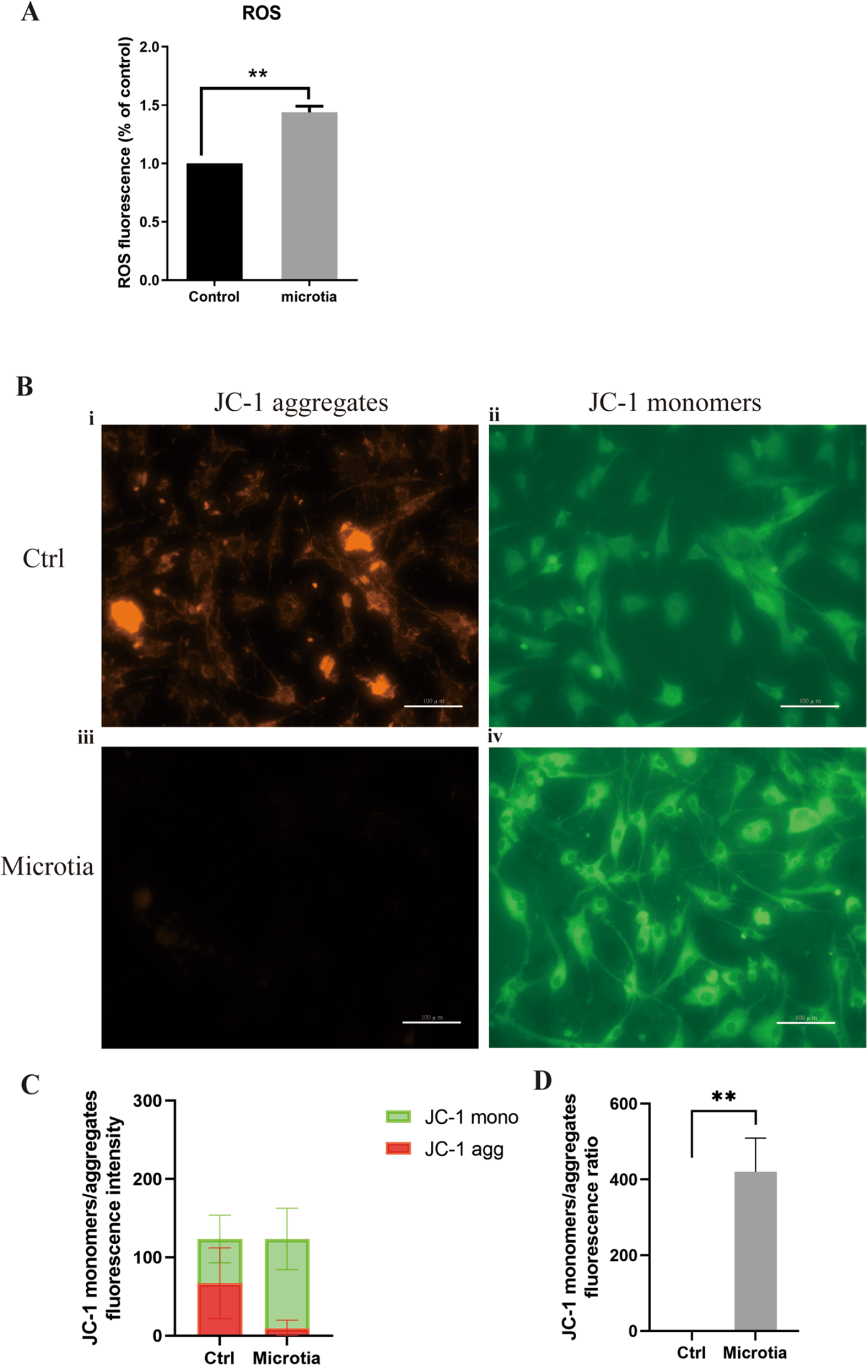
ROS Detection and Mitochondrial Membrane Potential (∆Ψm) Detection in Microtia Chondrocytes. **A** ROS levels of chondrocytes in patients with microtia in control cells. **B**: mitochondrial membrane potential (MMP/ΔΨm) based on JC-1 staining. **C** MMP fluoresence intensity. MMP is significantly lower in microtia chondrocytes compared to the ctrl group while maintaining a balance in healthy chondrocytes. **D** The monomers/aggregates fluorescence ratio of normal and microtia chondrocytes. The mitochondrial membrane potential in microtia chondrocytes was significantly lower than that of normal ear chondrocytes. Error bars represent the mean ± SD of three independent experiments. *p < 0.05, **p < 0.01

As we mentioned above, both WES and proteomic results indicate that cellular redox homeostasis may play a role in microtia, therefore, we test this hypothesis by ROS experiment and JC-1 experiment. Chondrocytes from different microtia patients were used as the experimental group, normal people’s ear chondrocytes were the control group and the positive control group was stimulated with 1ug/ well Rosup for 20 minutes. After detecting fluorescence intensity, ROS levels in the microtia group were significantly higher than those in the control group (p < 0.01) (Fig. 7A).

As mitochondria are the predominant source of ROS production, we measured mitochondrial membrane potential (MMP/ΔΨm) based on JC-1 staining (Fig. 7B). Red fluorescence represented a potential-dependent aggregation in the mitochondria, while green fluorescence represented the monomeric form of JC-1, appearing in the cytosol after mitochondrial membrane depolarization. We found that MMP was significantly lower in microtia chondrocytes compared to the ctrl group, moreover, ΔΨm maintained a balance in healthy chondrocytes (Fig. 7C). Our results showed that the green/red fluorescence ratio increased in the microtia group. In normal cells, JC-1 aggregated in mitochondria and the ratio was 0.91 ± 0.16. Microtia chondrocytes showed significantly a higher ratio (420.30 ± 154.60, *P* < 0.01) because the monomeric form JC-1 appeared in the cytosol, which indicated the dissipation of ΔΨm (Fig. 7D). These results collectively support the hypothesis that oxidative stress exists in microtia patients and may affect the mitochondrial function in chondrocytes.

## Discussion

Microtia is one of the leading congenital malformations in the plastic surgery field, it brings harm to children's psychology, and puts pressure on their families and increases society's burden, experts are attaching increasing importance to the exploration of microtia. One major obstacle in treating microtia is that the underlying mechanism is complicated and not clearly understood. Owing to the fact that auricular cartilage originates from NCCs [10], therefore, previous reports about microtia mainly focused on the development of NCCs. In this study, we further investigated the etiology of microtia accompanied by costal chondrodysplasia and sternal bone deformity by bioinformatic tools. WES and label-free analysis were conducted between the family members. Interestingly enough, both WES and proteomic results indicated that metabolic program, associated immune responses, and inflammatory and cellular redox homeostasis may play a pathological or at least a modulating role in microtia, and this has never been reported before.

The candidate genes *SOD3* and *PLG* by ACMG classification overlap with the proteomic results. PLG not only regulates coagulation and complement pathway, it is also responsible for neutrophil apoptosis and efferocytosis in inflammation, and functions as apolipoprotein binding [11], whereas in our case, it is downregulated in microtia patients when compared to his mother but upregulated than his father. It is widely believed that excessive reactive oxygen species (ROS) can lead to oxidative stress and DNA damage which can decrease MSCs self-renewing and multidirectional differentiation [12]. SOD3 protects cells from the toxic effect of RO intermediates by converting superoxide radicals into hydrogen peroxide and oxygen. Nightingale et al. found that decreased SOD3 level following BMSCs chondrogenesis [13]. Gavriilidis et al. knocked down SOD2 in chondrocytes, resulting in increased ROS levels, mitochondrial DNA strand breaks and decreased antioxidant capacity, and this may be a potential contributor to osteoarthritis [14].

The variant identified in the *PRKDC* gene is predicted to be pathogenic according to ACMG, it can recognize and repair DNA double-strand breaks [15], and interacts with autoimmune regulator (AIRE) to regulate B cells and natural killer (NK) cells, causing inflammation and immune dysregulation, and downstream S100A8 expression [16]. Plus, PRKDC is also involved in nervous system development [17]. Although S100A8 does not show significance in WES, it is upregulated in proteomics results. S100A8 is a calcium- and zinc-binding protein that plays a prominent role in the regulation of inflammatory processes and immune response. S100A8/A9 promotes cell death via autophagy and apoptosis and this occurs through the crosstalk of mitochondria and lysosomes via ROS [18]. The S100 family is closely related to chondrogenesis [19], and S100A8 and S100A9 up-regulate inflammatory cytokines through TLR-4 And MMP-1, -3, -9, and -13 to destroy bone [20].

*TASP1* encodes taspase 1(threonine aspartase 1, TASP1). TASP1 proenzyme intramolecularly proteolyzes and generates an active N-terminal 28 kDa α subunit and a 22 kDa C-terminal β subunit heterodimer whereas the β subunit is the active site for cleavage activity [21]. TASP1 cleaves various nuclear factors after an aspartate with substrates including the histone-methyltransferase mixed lineage leukemia 1 (MLL1/KMT2B), transcription factor IIA (TFIIAα-β/GTF2A1), TFIIA-like factor (ALFα-β) and so on [22]. TASP1 activateing MML1 is crucial for *HOX* and cyclin genes expression that participate in body segmentation and cell proliferation [23] and TASP1 cleaving TFIIAα-β make it more susceptible to degradation impedes its transcriptional activity in embryonal cell proliferation and morphogenesis. Tasp1-/- mice show craniofacial malformations [24] and facial features containing low-set ears [25] and TASP1 mutation has been reported in complex syndromes manifesting with facial and skeletal abnormalities [26]. As mitochondrial redox status can affect SAM level to modify histone methylation [27], and studies have identified SET/MLL as redox sensitive units of the H3K4me3 [28]. The relationship between Taspases and oxidative stress-mediated alterations in DNA/histone methylation and other post-translational modifications is worth to be explored in future studies.

The enrichment analysis of differently expressed genes (DEGs) revealed that mutated genes are majorly enriched in the cellular metabolic process, embryonic development process, oxidative stress and cytoplasmic vesicles, which are concordant with the results in proteomics analysis. It may reflect that metabolism may play an important regulating role in the pathogenesis of microtia. Expression differences could be a sign of the biochemical or physiological changes that might lead to microtia. Many researches have proved that immunometabolism is closely related to the development of diseases [29]. Oxidized low-density lipoprotein can trigger strong proinflammatory responses that can potentially contribute to the development of atherosclerosis [30].

What gives us implications in our study is the upregulated proteins in three groups are closely related to the immune system and oxidative stress. The highest change of protein is ORM2, which is reported to modulate the activity of the immune response and the complement cascades pathway [31]. ORM2 is at the same time a suppressor of Fe-related ROS [32]. SAA1 is also a major acute-phase inflammatory protein [33], it contributes to bone and cartilage destruction [34]. IGHD as its name implies, mediate the effector phase of humoral immunity [35]. Polymeric immunoglobulin receptor (PIGR) specifically binds to J-chain-containing secretory IgM and IgA, therefore, it is a critical protein in the mucosal immunity [36]. Vertebrate haemoglobin, a heterotetramer of paralogous α- and β-subunits that mediate respiratory oxygen transport and exchange [37], and can be cleared by binding to the haptoglobin (HP), in this way to scavenging HBB-mediated toxic effects caused by free radical and participates in the complementary pathways [38].

When comparing the proteomics results to the WES SNPs genes, there are overlapped results: MASP2 and FCN2, which are all belong to key proteins of the lectin pathway of complement. Ficolin 2 is the initiator and their N-terminal collagen-like domain interacts and forms complexes with the MASPs, which leads to the C4 and C2 cleavage and thus activation of the downstream complement cascade [39]. In our results, FCN2 is at least fivefold higher, and the variation is heterogenous in his mother. MASP2 is also at least fivefold higher and the variation is heterogenous in the patient’s sister and father, both are in the same trend with the proteomics results. It is reported that a higher level of ficolin is related to autoimmune disorders [40]. There have been reports about complement-associated and prostaglandin-dependent bone metabolism and physiologic remodeling [41], such as axial spondyloarthritis [42]. It is also believed that complement activation can release cytokines, proteases, and ROS, thereby contributing to inflammation and generates deleterious effects [41]. But so far, none of these molecules have been reported in the pathology of microtia.

Cadherin-13 (CDH13) that repeat in the groups A vs. B and A vs. D is an important adhesion molecule that mediates cell–cell adhesion in neural tube development [43], while there is a report about CDH13 and CRTAC expression in the limb mesenchyme control contrarily proprioceptor identity [44]. Coincidently, CRTAC is downregulated in our WES result. Cdh13 protects against cardiac stress through its association with adiponectin through the AMPK signaling pathway in mice [45]. What is more, research has proved that, Cdh13 serves as an anchor for tethering of adiponectin to M2 macrophages and can promote the cell proliferation by activation of Akt [46]. In our proteomic results, CDH13, adiponectin and CRTAC are all downregulated, which may imply the disruption of the cell proliferation and neural tube development, and inflammation may participate in the malfunction.

Besides, the commonly dysregulated genes were significantly enriched in GO terms in Fig. 3 and Table 9 shows that most proteins are significantly enriched in lipid metabolic process, peroxidase activity, and the hemoglobin related functions. LEPR^+^ MSCs give rise to most bone and adipocytes in adult bone marrow and can form bony ossicles that support hematopoiesis in vivo [47]. ApoE is involved in lipoprotein metabolism and ApoE -/- mice showed intervertebral disc degeneration partially due to increased matrix metalloproteinase (MMP) -3, -9, and -13 levels [48]. In addition, Farnaghi et al*.* found that hypercholesterolemia caused the degeneration of articular disc cartilage in osteoarthritis by the production of mitochondrion-derived oxidative stress [49]. Evidence shows that there is a link between oxidative stress and cartilage degradation.

What is more, emerging evidence has shown that cell metabolism influences gene expression by modifying the epigenome, which can regulate stem cell pluripotency, differentiation and somatic cell reprogramming [50]. ALDOB participates in fructose and other central carbon metabolism by reversibly cleaving fructose 1,6-diphosphate into glyceraldehyde 3-phosphate (G3P) and dihydroxyacetone phosphate (DHAP) [51]. Lipogenesis requires oxidative phosphorylation to provide a large amount of energy for lipid storage, while chondrocytes mainly rely on glycolysis for energy supply [52]. We hypothesize that reduced ALDOB protein levels in microtia patients may lead to decreased glycolysis and decreased MSC’s chondrogenic ability in some way which would be studied in future studies.

**Fig. 8:**
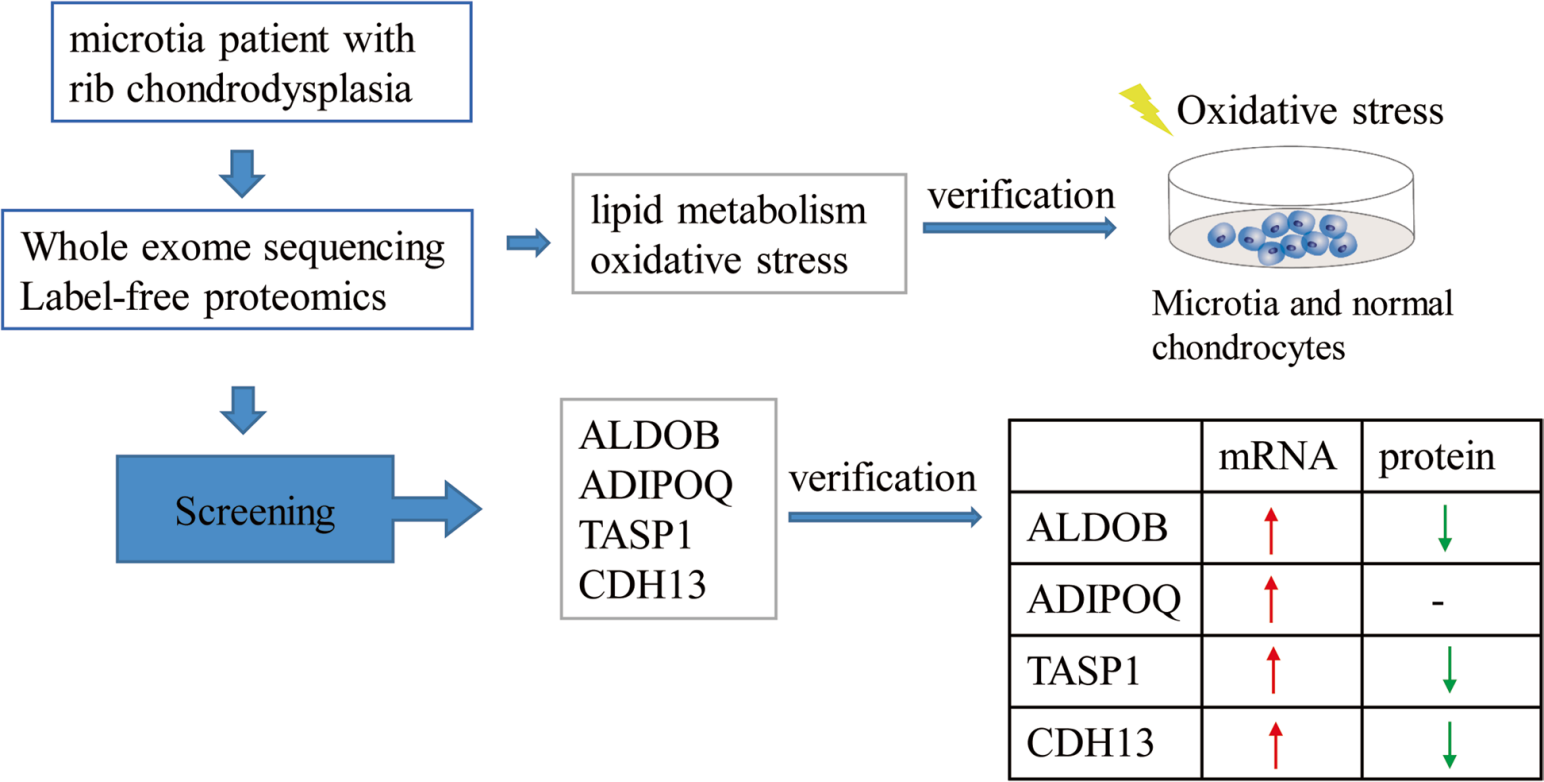
Graphical Abstract. A graphical overview of this study

In label-free proteomics analysis, ALDOB protein is 5.41239 times higher in the patient compared to his sister(B) and 3.87038 times higher when compared to the mother in the A/C group, CDH13 and ADIPOQ are 0.35024 and 0.26241 times in A vs. B group respectively and show the same trend in A vs. D group by 0.31112 and 0.29045 time respectively. This is coherent with the result in IHC that CDH13 and ADIPOQ are lower in microtia patients. But the real-time PCR of other microtia patients shows that mRNA levels of ALDOB, ADIPOQ, CDH13, and TASP1 are all higher in the microtia group while IHC results show all of the four proteins are significantly lower when compared to the control group as shown in Figs. 7 and 8. This is probably due to the translational modifications of the mRNAs and shall be further explored as a candidate disease mechanism.

The collective findings provide novel insights into the pathogenesis of congenital microtia-atresia. It can be seen that the screened proteins are mainly involved in lipid metabolism, immunology, and inflammation process, and our in vitro verifications have proved that ROS do exist in microtia patients, as some fault programmed gene expression and misregulated microenvironment during the embryonic stage could be kept in the entirety and outer ear keeps on developing after birth [53], therefore these faults may affect ear length, width, prominence and degree of auricular development. The relationships between oxidative stress and these proteins in cancers or inflammatory processes have been widely studied [29], but the relationship between oxidative stress and chondrogenic defect of microtia has not been thoroughly realized. Alterations of the redox state in microtia may provide new insight into pathogenic mechanisms and refine therapeutic strategy, with the ultimate goal of improving the quality of life for the patients.

### Potential limitations

There are also some limits of proteomic analyses as protein may not fully reflect the genetic variants and can only show the pathology of micro-environmental effect in this case. To overcome this limitation, we use the whole-exome sequencing to find out the possible variations in microtia at the genome level. However, the exome region constitutes only 1–2% of the entire genome, and WES inevitably leaves out the non-coding mRNA elements and/or intronic or intergenic regulatory regions that may be responsible for the disease phenotypes [2]. What is more, due to individual variances, more microtia patients are needed to further verify our findings. Last but not least, more explorations of how oxidative stress and malfunction of genes related to embryonic development in vivo shall be done in our future studies.

## Conclusion

In summary, based on comprehensive analyses of WES from the patient with microtia and costal chondrocyte dysplasia, the present study found out SNPs including *PRKDC*, *LEPR*, *PLG*, MUC6, *DGKK*, *GPR161*, *WDR36*, *BPTF*, *LRBA*, *SOD3*, *KCNQ4* and de novo CNVs in *ESPNP* may be the candidate genes that may function at the onset of the disease. Besides, there are likely deleterious CNVs including *ASMT*, *DHRSX*, *ASMTL*, *AKAP17A*, and *P2RY8*. Multi-omics data find out proteins with lipid metabolism, immune response, the complement cascades pathway and oxidative stress may take part in the pathogenesis of this rare case of congenital microtia. The verification of the multi-omics results with other microtia patients identified that ALDOB, ADIPOQ, CDH13 and TASP1, and oxidative stress may play a role in chondrogenic anomalies. In view of this, we speculate the importance of the interrelationship between gene network and environmental factors on the disease. Up till now, there is no further study examining the ROS pathogenic mechanism involved in microtia. A deeper understanding of microtia etiology will not only shed light on the occurrence of this defect, but also facilitate better prevention and therapeutic strategies for microtia.

## Methods

### Patients and ethics

A patient showing congenital microtia-atresia was hospitalized at The First Affiliated Hospital of Xi'an Jiao Tong University. Family history was obtained and clinical features of all the study subjects and evaluated via a review of medical records. This study was approved by the ethics committee of the First Affiliated Hospital of Xi'an Jiao Tong University Medical College and a written informed consent was provided. Authorization was also obtained for the disclosure of recognizable individuals in photographs and the collection of blood for further analyses.

### WES

Whole blood (3 ml) was obtained from each individual. Genomic DNA extracted from peripheral blood for each sample was fragmented to an average size of 180 ~ 280bp and subjected to DNA library creation using established Illumina paired-end protocols. Whole-exome capture was performed using an Agilent SureSelect Human All Exon V6 enrichment capture kit (Agilent Technologies, Santa Clara, CA, USA). The Illumina Novaseq 6000 platform (Illumina Inc., San Diego, CA, USA) was utilized for genomic DNA sequencing in Genechem Bioinformatics Technology Co., Ltd (Beijing, China) to generate 150-bp paired-end reads with a minimum coverage of 10 × for ~ 99% of the genome (mean coverage of 100 ×). After sequencing, basecall files conversion and demultiplexing were performed with bcl2fastq software (Illumina). The resulting FASTQ data were submitted to in-house quality control software for removing low quality reads, and then were aligned to the reference human genome (hs37d5) using the Burrows-Wheeler Aligner (bwa), and duplicate reads were marked using SAMtools. SNP/INDEL calling: Single nucleotide variants (SNVs) and indels were called with samtools to generate gVCF. The raw calls of SNVs and INDELs were further filtered with the following inclusion thresholds: 1) read depth > 4; 2) Root-Mean-Square mapping quality of covering reads > 30; 3) the variant quality score > 20. CoNIFER (V0.2.2) was used to detect CNVs. Annotation was performed using ANNOVAR (2017 June 8). Annotations included minor allele frequencies from public control data sets as well as deleteriousness and conservation scores enabling further filtering and assessment of the likely pathogenicity of variants. The classification system of the American College of Medical Genetics and Genomics (ACMG) was used to predict the harmfulness of variations.

### Label-free proteomics

Serum pools were depleted of the most abundant proteins from each individual using the Agilent Human 14 Multiple Affinity Removal System Column following the manufacturer’s protocol (Agilent Technologies). The 10 kDa ultrafiltration tube (Sartorius) was used for desalination and concentration of low-abundance components. One volume of SDT buffer (4%SDS, 100 mM Tris–HCl, pH 7.6) was added, boiled for 15 min and centrifuged at 14000 g for 20 min. The supernatant was quantified with the BCA Protein Assay Kit (Bio-Rad, USA). The sample was stored at -20 °C. 20 µg of proteins for each sample were mixed with 6X loading buffer respectively and boiled for 5 min. The proteins were then separated on 12.5% SDS-PAGE gel. Protein bands were visualized by Coomassie Blue R-250 staining. 200 μg of proteins for each sample went filter-aided sample preparation (FASP digestion) and LC–MS/MS analysis was performed on a Q Exactive Plus mass spectrometer (Thermo Fisher Scientific) that was coupled to Easy nLC (Thermo Fisher Scientific). 2μg peptide was loaded onto the C18-reversed phase analytical column (Thermo Fisher Scientific, Acclaim PepMap RSLC 50um X 15 cm, nano viper, P/N164943) in buffer A (0.1% formic acid) and separated with a linear gradient of buffer B (80% acetonitrile and 0.1% Formic acid) at a flow rate of 300 nl/min. The linear gradient was as follows: 5% buffer B for 5 min, 5–28% buffer B for 90 min, 28–38% buffer B for 15 min,38–100% buffer B for 5 min, hold in 100% buffer B for 5 min. MS data was acquired using a data-dependent top10 method dynamically choosing the most abundant precursor ions from the survey scan (350–1800 m/z) for HCD fragmentation. MS1 scans were acquired at a resolution of 70,000 at m/z 200 with an AGC target of 3e6 and a maxIT of 50 ms. MS2 scans were acquired at a resolution of 17,500 at m/z 200 with an AGC target of 2e5 and a maxIT of 45 ms, and the isolation width was 2 m/z. Only ions with a charge state between 2–6 and a minimum intensity of 2e3 were selected for fragmentation. Dynamic exclusion for selected ions was 30 s. The normalized collision energy was 27 eV. The MS data were analyzed using MaxQuant software version 1.6.14.0. MS data were searched against the Uniprot_HomoSapiens_20386_20180905 database, downloaded on http://www.uniprot.org/. An initial search was set at a precursor mass window of 6 ppm. The search followed an enzymatic cleavage rule of Trypsin/P and allowed maximal two missed cleavage sites and a mass tolerance of 20 ppm for fragment ions. Carbamidomethylation of cysteines was defined as fixed modification, while protein N-terminal acetylation and methionine oxidation were defined as variable modifications for database searching. The cutoff of the global false discovery rate (FDR) for peptide and protein identification was set to 0.01. Protein abundance was calculated on the basis of the normalized spectral protein intensity (LFQ intensity). |log2fold change (FC) |≥ 1 and p value < 0.05(Student’s test) were selected as the threshold for screening.

### Real-time polymerase chain reaction (PCR)

Total RNA was extracted using TRIzol reagent (Invitrogen, USA). The total RNA was reversely transcribed using oligo dT (Promega, USA), M-MLV reverse transcriptase (Promega, USA) and dNTPs (Sigma–Aldrich, Germany) according to the manufacturer’s instructions. Real-time PCR was performed with a Light Cycler 480 SYBR Green Master and Light Cycler 96 system (Roche, Switzerland) according to the manufacturer’s instructions. Primers were designed by Primer Premier 5 software (Canada) and are listed in the Supplementary Table S5. The relative expression of each gene was normalized by comparison with the reference GAPDH and measured with the 2-△△CT method.

### Immunohistochemical staining

Samples from the patients’ and normal people’s cartilage pieces were fixed in 4% paraformaldehyde for 24 h prior to embedding in paraffin. The samples were sectioned into 5-μm slices, mounted on glass slides, and stained with hematoxylin and eosin (HE). The primary antibodies used were mouse anti-human monoclonal antibody (ADIPOQ 1:200, 21,613–1-AP; ALDOB 1:200,18,065–1-AP; CDH13 1:50, 12,618–1-AP; TASP11:50, 16,739–1-AP; all from Proteintech, China), followed by incubation of biotin-labeled second antibody(Cat# kit-9730; MXB, Fuzhou, China) at RT for 50 min followed by enzyme-conjugated streptavidin (Cat# kit-9730; MXB, Fuzhou, China) incubate at RT for 50 min. Develop color with 3,3’-diaminobenzidine (Cat# DAB-1031; MXB, Fuzhou, China) and wash in water. Finally, the sections are stained with hematoxylin staining. The slides were then examined and photographed using the Leica SCN400 slide scanner and the integrated density was analyzed by Image-Pro Plus 6.0 software.

### Cell culture

Auricular cartilage of microtia patients or normal people who had done rhinoplastic surgery was washed in PBS + 100 U/mL penicillin G, 100 μg/mL streptomycin (Gibco, Gaithersburg, MD, USA) and fragmented into 1mm^3^ pieces in 0.2% type IV collagenase (Gibco, Gaithersburg, MD, USA) and digested overnight at room temperature. The cell suspension was centrifuged at 1000 rpm for 5 min and filtered by 70 μm mesh (BD, USA) and cultured in the complete medium consisted of DMEM (Gibco, Gaithersburg, MD, USA) containing 10% heat-inactivated fetal bovine serum (BIOEXPLORER, USA). Chondrocytes were maintained in a humidified incubator containing 5% CO^2^ at 37 °C and the first or second passages were used for the following experiments.

### ROS analysis

Intracellular ROS levels were measured using 2’,7’-dichlorofluorescin diacetate (DCFH-DA; Cat#S3300S, Beyotime, China) according to the manufacturer’s instructions. Briefly, ear chondrocytes of microtia patients, normal people, ALDOB^OX^ and ALDOB^pEN^ groups were seeded to 48-well plates. Rosup was added as positive control, and one well was left as the blank control. DCFH-DA/Rosup was diluted with the serum-free medium at 1:1000 to achieve a final concentration of 10 µM and were incubated with cells for 30 min at 37 °C in the dark. The cells were washed 3 times with PBS to fully remove the DCFH-DA that did not enter the cells. The fluorescence intensity was detected with the excitation wavelength of 488 nm and the emission wavelength of 525 nm using the microplate reader (Cytation 5 M, Bio-Tek, USA).

### Mitochondrial membrane potential detected by JC-1

Mitochondrial transmembrane potential (Δ Ψ m) was detected using the JC-1 mitochondrial membrane potential assay kit (Cat#C2005, Beyotime Biotech, China), following the manufacturer’s protocol. Cells cultured in 6-well plates were incubated with 1 ml JC-1 staining solution (10 µg/mL) at 37℃ for 20 min, and then the supernatant was removed and washed twice with JC-1 dyeing buffer (1X). Loss of mitochondrial membrane potential (Δ Ψ m) was assessed by fluorescence microscope (Axio Observer 7, Zeiss, German).

### Statistics analysis

Results are expressed as the mean ± SD (standard deviation) with at least 3 independent biological repeats. The unpaired, two-tailed Student’s *t*-test was used to analyze the difference between groups using Prism 6 (GraphPad) software. *P* < 0.05 was considered as significant.

### Supplementary Information

**Supplementary Material 1.**

**Supplementary Material 2.**

**Supplementary Material 3.**

**Supplementary Material 4.**

**Supplementary Material 5.**

**Supplementary Material 6.**

## Data Availability

All data can be download on the journal as of the date of publication. The data presented in this study are available on request from the corresponding author due to data privacy protection.

## Abbreviations

ACMG: American Society for Medical Genetics and Genomics
ADIPOQ: Adiponectin
AIRE: Autoimmune regulator
AKAP17A: A-kinase anchoring protein 17A
ALDOB: Fructose-bisphosphate aldolase B
ALS: Amyotrophic lateral sclerosis
APOA2: Apolipoprotein A-II
ASMT: Acetyl serotonin methyltransferase
ASMTL: Acetyl serotonin O-methyltransferase like
BMPs: Bone morphogenetic proteins
BP: Biological processes
CC: Cellular component
CDH13: Cadherin-13
CDH13: Cadherin-13
CNVs: Copy number variations
DEGs: Differently expressed genes
DFNA2: Deafness nonsyndromic autosomal dominant 2
DHRSX: Dehydrogenase/reductase X-linked
EFEMP1: EGF-containing fibulin-like extracellular matrix protein 1
F13A1: Coagulation factor XIII A chain
FCN2: Collagen/fibrinogen domain-containing lectin2
FDR: False discovery rate
FGFs: Fibroblast growth factors
GO: Gene Ontology
GPCR: G-protein-coupled receptor
GPR161: G protein-coupled receptor 161
GSC: Goosecoid gene
HBB: Hemoglobin subunit beta
HP: Haptoglobin
HP: Haptoglobin
IGA2: Immunoglobulin alpha-2 heavy chain
IGHD: Immunoglobulin delta heavy chain
InDel: Insert and deletion
ITGB1: Integrin beta 1
KCNQ4: Potassium voltage-gated channel, KQT-like subfamily, member 4
LEPR: Leptin receptor
LRBA: Lipopolysaccharide-responsive, beige-like anchor protein
MASP2: Mannan-binding lectin serine protease 2
MCAM: Cell surface glycoprotein MUC18
MF: Molecular function
MLL1: Methyltransferase mixed lineage leukemia 1
MMP: Matrix metalloproteinase
MSCs: Mesenchymal stem cells.
NCCs: Neural crest cells
NK: Natural killer
ORM2: Alpha-1-acid glycoprotein 2
P2RY8: P2Y receptor family member 8
PIGR: Polymeric immunoglobulin receptor
PLG: Plasminogen
PRDX2: Peroxiredoxin-2
PRKDC: Protein kinase, DNA-activated, catalytic polypeptide
ROS: Reactive oxygen species
S100A8: Protein S100-A8
SAA1: Serum amyloid A-1 protein
SERPINA1: Alpha-1-antitrypsin
SHBG: Sex hormone-binding globulin
Shh: Sonic hedgehog
SNPs: Single nucleotide polymorphisms
SNVs: Single nucleotide variants
SOD3: Superoxide dismutase-3
TASP1: Threonine aspartase
TF: Serotransferrin
UNC45: Hyperradiosensitivity of murine SCID mutation, complementing-1
UNC45B: C. elegans, homolog of, B
VNN1: Pantetheinase
WDR36: WD repeat-containing protein 36
WES: Whole-exome sequence
WNTs: Wingless/INT
