# Supplementary figures and images for: Multi-omics analysis of a case of congenital microtia reveals aldob and oxidative stress associated with microtia etiology
Source: Orphanet J Rare Dis. 2024 May 27;19:218. doi: 10.1186/s13023-024-03149-2 (PMC11129396; doi:10.1186/s13023-024-03149-2)

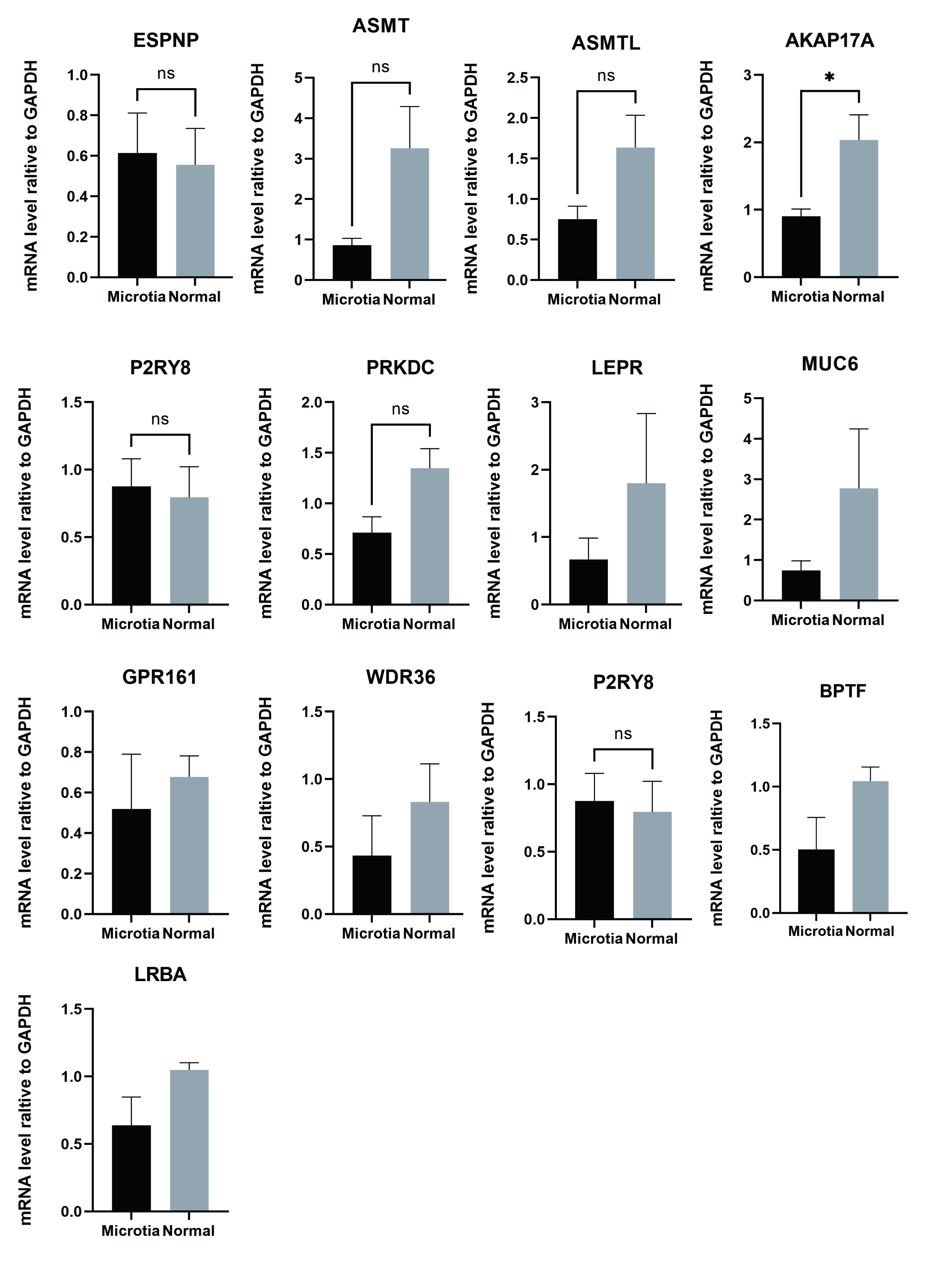

Supplement: Supplementary file 5 — Supplementary Material 5. [file 13023_2024_3149_MOESM5_ESM.tif]
